# Bryophyte Species Richness and Composition along an Altitudinal Gradient in Gongga Mountain, China

**DOI:** 10.1371/journal.pone.0058131

**Published:** 2013-03-05

**Authors:** Shou-Qin Sun, Yan-Hong Wu, Gen-Xu Wang, Jun Zhou, Dong Yu, Hai-Jian Bing, Ji Luo

**Affiliations:** Key Laboratory of Mountain Surface Processes and Ecological Regulation, Institute of Mountain Hazards and Environment, Chinese Academy of Sciences, Chengdu, China; DOE Pacific Northwest National Laboratory, United States of America

## Abstract

An investigation of terrestrial bryophyte species diversity and community structure along an altitudinal gradient from 2,001 to 4,221 m a.s.l. in Gongga Mountain in Sichuan, China was carried out in June 2010. Factors which might affect bryophyte species composition and diversity, including climate, elevation, slope, depth of litter, vegetation type, soil pH and soil Eh, were examined to understand the altitudinal feature of bryophyte distribution. A total of 14 representative elevations were chosen along an altitudinal gradient, with study sites at each elevation chosen according to habitat type (forests, grasslands) and accessibility. At each elevation, three 100 m × 2 m transects that are 50 m apart were set along the contour line, and three 50 cm × 50 cm quadrats were set along each transect at an interval of 30 m. Species diversity, cover, biomass, and thickness of terrestrial bryophytes were examined. A total of 165 species, including 42 liverworts and 123 mosses, are recorded in Gongga mountain. Ground bryophyte species richness does not show any clear elevation trend. The terrestrial bryophyte cover increases with elevation. The terrestrial bryophyte biomass and thickness display a clear humped relationship with the elevation, with the maximum around 3,758 m. At this altitude, biomass is 700.3 g m^−2^ and the maximum thickness is 8 cm. Bryophyte distribution is primarily associated with the depth of litter, the air temperature and the precipitation. Further studies are necessary to include other epiphytes types and vascular vegetation in a larger altitudinal range.

## Introduction

Bryophytes are an important component of ecosystem biodiversity and make up a significant part of species richness [1]–[3] and plant biomass in forests in some cases [4], [5]. They also play a prominent role in ecosystem functions, such as soil development [6], [7], nutrient biogeochemical cycling [4], [8], water retention [9], plant colonization [10], seed germination, seedling growth, and forest renovation [5], [8],[11]. However, bryophytes are still rarely considered in biodiversity surveys when compared with their vasular counterparts [12]. The reasons include difficulties in identification, fewer specialists, less literatures on bryophyte taxonomy in tropical areas, and the high-costs (both time and money) for searching and identifying bryophytes. Bryophytes have wider distribution and longer altitudinal gradient than vascular plants, and thus strong generalizations on observable changes in diversity along latitudinal and altitudinal gradients can be made according to bryophyte distribution if any patterns do exist [13]. They therefore have been deemed as ideal candidates for altitudinal studies in recent years [13]. With growing interest in climate change, using bryophytes as indicator species for climate change has also attracted more attention due to their sensitivity to environmental change [14]. Research on bryophyte diversity, richness and distribution is therefore increasing [12], [15].

Several altitudinal patterns of bryophyte richness and distribution have been reported, such as decreasing [16] or increasing [12], [17]–[19] with elevation increasing, a hump-shaped distribution [15] and no obvious trends at all [13]. Although there still is no explanation for these differences, it is now widely accepted that peak diversity coincides with optimum environmental conditions [12]. However, various factors such as forest properties include stand structure [20], canopy opening [21], [22], forest management [23], and climate [24], [25] can cause variation in species richness, growth rate, and community structure of bryophytes.

Much research has been conducted to find out the main factors influencing bryophyte richness and distribution pattern. Pharo and Beattie [26] discovered the important role of substrate on bryophyte diversity and composition. Andrew et al. [13] pointed out that altitudinal gradient may control community structure and diversity. Meanwhile, they suggested that the factors operating at smaller scales (moisture and microhabitats) should be studied to understand the underlying mechanisms. Frahm and Ohlemüller [17] found that liverwort species increase with increasing humidity, cloud cover, and mist. Some researchers indicated that bryophyte distribution is primarily influenced by macroclimatic factors (such as rainfall and temperature) [27], and microenvironment features of shade (light intensity), habitat humidity and temperature [28]. Batty et al. [29] regarded that site factors, such as age, forest composition, moisture regime, and substrate characteristics (e.g., pH and nutrient status), controll the bryophyte distribution. Vellak et al. [30] inferred that tree layer, especially the distance to the nearest tree, is the dominant factor that influences the diversity and distribution of ground and field-layer species.

Views about the factors mainly influencing bryophyte growth and distribution are still debated. The ecological mechanisms of bryophyte richness and distribution pattern along altitudinal gradients still need to be further investigated. In addition, the biomass, thickness and cover of bryophytes are key indices reflecting bryophyte growth status. Such data are useful for modeling whole ecosystem response to climate change, modeling ecosystem carbon and nutrient cycling, and improving our understanding of the ecological roles of bryophytes in forest ecosystem. However, most of the previous research did not consider the biomass, thickness and cover of bryophytes.

The objectives of the present study were (1) to describe the distribution pattern of terrestrial bryophytes along an altitudinal gradient in Gongga Mountain, Sichuan, China, and (2) to find out the major factors which influence bryophyte diversity and distribution. This investigation will be helpful in identifying strategies and opportunities for the conservation of bryophyte species, and would also be the basis of climate change research in this area. We hypothesized that climate is the main factor that influences bryophyte distribution.

## Methods

### Study Area

The study was conducted in Gongga Mountain (29°20′ - 30°20′ N, 101°30′ - 102°15′ E), which is located in Sichuan, southwestern China, at the eastern edge of the Tibetan Plateau. The peak of Gongga Mountain is 7556 m a.s.l., the summit in the Hengduan Mountain Range. Within a horizontal distance of 28 km the relative height drops 6,400 m to the eastern bottom of Gongga Mountain. On the eastern slope of Gongga Mountain, the annual precipitation varies between 1,068 and 3,210 mm (increases with the elevation below 3,650 m a.s.l., but decreases with the elevation above 3,650 m a.s.l.), the mean annual temperature is between −2.6 and 14.5°C depending on the altitude. The mountain has an intact vertical zonality from subtropical vegetation to alpine cold vegetation [31]. The vegetation in the study area varies from evergreen broad-leaved forest (1,600–2,200 m a.s.l.), mixed evergreen and deciduous broad-leaved forest (2,200–2,400 m a.s.l.), mixed broadleaf-conifer forest (2,400–2,800 m a.s.l.), dark coniferous forest (2,800–3,600 m a.s.l.), alpine shrubland (3,600–4,000 m a.s.l.), alpine meadow (4,000–4,600 m a.s.l.), alpine sparse vegetation (4,600–4,900 m a.s.l.), to ice covered area (above 4900 m a.s.l.).

### Sampling

**Figure 1:**
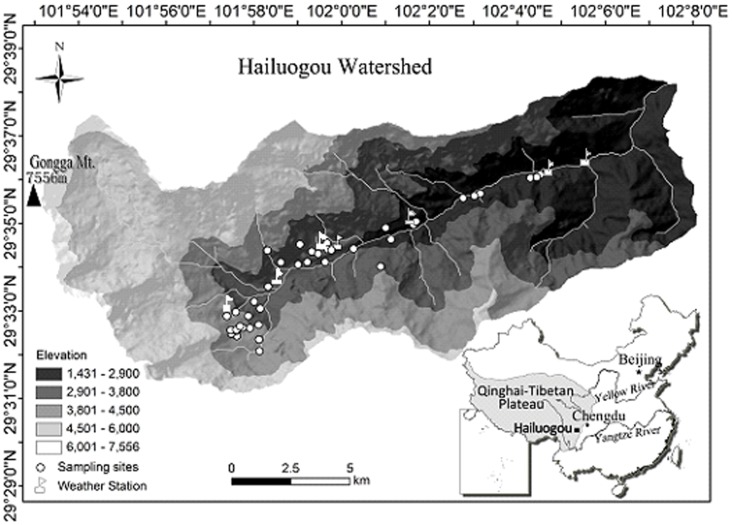
Sampling sites.

In June 2010, bryophytes were collected along an altitudinal transect between 2,001 and 4,221 m a.s.l. All permits required to carry out the field studies were obtained from the Natural Park authorities. A total of 14 sites at representative elevations were chosen along the altitudinal gradient, with each site chosen according to habitat type (forests, grasslands) and accessibility (Figure 1). At each site, three 100 m × 2 m transects with 50 m apart were set along the contour line. All the three transects of one elevation level were homogenous and comparable to each other with regard to forest type, herb layer, forest management, soil type, and climate. In each transect, three 50 cm × 50 cm quadrats were set in the center with an interval of 30 m along the horizontal distances. If large rocks or dead wood occurred in a transect, the quadrat was moved to the nearest suitable place and established.

A screen with 400 grids (2.5 cm × 2.5 cm) was then placed on each quadrat. The percentage cover of the whole ground floor bryophytes was recorded based on the number and space of grids occupied with bryophytes [32]. The thickness of bryophyte layer was recorded at 5 points separately located in the middles of the four sides and in the center of each quadrat using a ruler with a millimeter scale. Bryophyte species found in each quadrat were collected, coded, and kept for proper identification. All bryophytes in each quadrat were then destructively collected and put in clean contamination-free polyethylene (PE) plastic bags. In the field, the PE bags were marked with the transect number. In the laboratory, the bryophytes were separated from other vegetation and washed with tap water to remove dirt. All samples were oven dried at 40°C, and then weighed to calculate the biomass. Collections made for identification of species were not included in the biomass measurements. This did not significantly affect the bryophyte biomass as the samples collected for species identification were very small, and the density of bryophytes was also very small. Bryophyte species identification was performed with a stereo microscope and a light microscope. The nomenclature was after literatures such as Flora Bryophytorum Sinicorum [33]–[41], Illustrations of Bryophytes of China [42], and two others [43], [44].

### Environmental and Climate Indices

Climate indices, including air temperature, relative humidity (RH), precipitation, soil temperature, and soil moisture in each sampling elevation, were obtained from meteorological stations set up by the Alpine Ecosystem Observation and Experiment Station of Gongga Mountain. There are seven meteorological stations along the altitudinal gradient. For sites without a meteorological station, the climatic conditions were estimated after adjusting the meteorological data from the closest meteorological station to consider the effect of altitude. To this end, air temperature gradient (0.5°C of decrease per 100 m altitude; *R^2^* = 0.9677, *P*<0.01), RH (1.35% of increase per 100 m altitude below 3,060 m a.s.l., *R^2^* = 0.9189, *P*<0.01; 1.58% of decrease per 100 m altitude above 3,060 m a.s.l., *R^2^* = 0.9992, *P*<0.01), precipitation (90.85 mm of increase per 100 m altitude below 3,650 m a.s.l., *R^2^* = 0.8181, *P*<0.01; 391.16 mm of decrease per 100 m altitude above 3,650 m a.s.l., *R^2^* = 1.0000, *P*<0.01), and soil temperature (0.5°C of decrease per 100 m altitude; *R^2^* = 0.8734, *P*<0.001) were calculated using the yearly climatic data of the seven meteorological stations.

Vegetation type, canopy height and closure, aspect, slope, and the geographic position were investigated in each transect. Canopy height was determined by measuring the height of one representative tree in each transect using a ruler and a tape measure [45]. The tree height was calculated based on the distance from the tree base to the observing site, and inclination angle of a line from the observing site to the tree top. Canopy closure was measured with a spherical densitometer at four randomly chosen spots and then averaged within each transect [45]. The depth of litter was measured at the same 5 points where bryophyte thickness was measured. Then the topsoil (0–5 cm) at the same 5 points was collected for soil pH and Eh (redox potential) measurement. The soil pH and Eh were measured in 1∶2.5 soil–H_2_O solutions using a pH/Eh meter (Thermo Electron Corporation, US). The environmental and climatic factors along the altitudinal gradient were presented in Table 1.

**Table 1:** Environmental and climatic factors along the altitudinal gradient.

Site No.	Transect No.	AL (m)	VT	AT (°C)	RH (%)	PR (mm)	ST (°C)	pH	Eh (S/cm)	LI (cm)	SL (°)	CC (%)	CH (m)	AS
1	1	2001	EBLF	10.47	80.74	1234	14.37	6.58	239±7.2	6.2±0.36	5	89±4.4	14±2.0	S
	2	2001	EBLF	10.47	80.74	1234	12.37	6.58	262±6.2	6±0.26	0	93±2.6	12.9±1.9	S
	3	2023	EBLF	10.36	81.04	1254	12.37	6.58	255±11.1	5.8±0.17	1	85±2.5	11.8±1.0	S
2	4	2301	EDBF	8.97	84.79	1506	12.26	6.76	288±10.6	3.4±0.44	1	88±3.6	13.3±0.8	E
	5	2301	EDBF	8.97	84.79	1506	10.87	6.76	293±6.1	3.7±0.17	1	89±4.4	15.6±0.7	E
	6	2359	EDBF	8.68	85.57	1559	10.87	6.76	310±5.6	3.4±0.26	15	84±1.7	13.1±0.6	E
3	7	2760	BLCF	7.56	94.71	1923	6.83	7.2	370±6.6	2.3±0.10	1	79±4.0	16.5±1.2	E
	8	2760	BLCF	7.56	94.71	1923	6.83	7.2	384±5.0	2±0.26	3	83±2.0	20±1.8	E
	9	2784	BLCF	6.55	91.31	1945	8.57	7.2	371±7.5	1.7±0.17	2	84±5.6	19.6±1.2	E
4	10	2964	DCF	5.65	93.74	2108	8.45	6.4	204±7.9	2.5±0.36	30	90±2.6	23±2.3	NW
	11	2964	DCF	5.65	93.74	2108	7.55	6.4	209±8.5	2.6±0.46	45	93±2.6	21.4±1.2	NW
	12	2964	DCF	5.65	93.74	2108	7.55	6.4	223±10.8	2.4±0.44	45	84±4.6	18±2.7	N
5	13	3044	DCF	5.25	94.82	2181	7.37	6.54	333±7.2	1.7±0.26	10	90±2.6	22.9±0.8	E
	14	3060	DCF	4.47	94.33	1933	5.01	6.54	342±8.9	1.7±0.20	5	93±1.7	19.3±1.8	E
	15	3060	DCF	4.47	94.33	1933	5.01	6.54	342±8.7	2±0.36	30	93±2.0	19.6±1.3	E
6	16	3103	DCF	4.22	94.19	2064	5.67	6.8	349±7.5	1.7±0.30	30	87±2.6	18.6±0.6	E
	17	3106	DCF	4.94	94.02	2237	6.86	6.8	358±11.5	2±0.20	25	89±4.4	20.9±0.6	E
	18	3106	DCF	4.94	94.02	2237	6.84	6.8	373±4.6	1.7±0.10	35	94±1.0	22.3±1.8	SE
7	19	3174	DCF	4.60	92.95	2299	6.84	5.66	209±7.0	2.6±0.26	62	71±2.6	19.4±0.9	E
	20	3174	DCF	4.60	92.95	2299	6.50	5.66	217±6.6	2.5±0.17	58	72±2.6	20.2±1.8	E
	21	3174	DCF	4.60	92.95	2299	6.50	5.66	228±5.6	2.4±0.17	60	76±3.6	21.6±1.4	E
8	22	3247	DCF	4.24	91.79	2366	6.50	6.89	399±10.6	2.6±0.26	61	76±2.6	18.3±0.4	SE
	23	3247	DCF	4.24	91.79	2366	6.14	6.89	415±6.1	2.6±0.53	61	76±3.5	20.2±0.7	SE
	24	3247	DCF	4.24	91.79	2366	6.14	6.89	401±6.7	2.3±0.31	58	82±3.6	22.4±1.5	SE
9	25	3650	APS	3.07	85.42	3211	5.40	6.63	340±7.8	0.1±0.1	2	0	0	E
	26	3650	APS	3.07	85.42	3211	5.40	6.63	335±7.9	0.2±0.1	15	0	0	E
	27	3650	APS	3.07	85.42	3211	5.40	6.63	357±9.6	0±0.00	13	0	0	E
10	28	3725	APS	1.85	84.24	2917	4.12	6.78	332±7.5	0±0.00	55	0	0	E
	29	3758	APS	1.68	83.71	2788	3.75	6.78	336±7.9	0.1±0.10	62	0	0	E
	30	3758	APS	1.68	83.71	2788	3.58	6.78	352±7.0	0.2±0.10	63	0	0	E
11	31	3817	APS	1.39	82.78	2557	3.58	6.83	232±6.6	1.9±0.30	56	0	0	W
	32	3817	APS	1.39	82.78	2557	3.29	6.83	257±9.6	1.9±0.10	63	0	0	W
	33	3817	APS	1.39	82.78	2557	3.29	6.83	252±12.8	1.6±0.26	61	0	0	NW
12	34	3987	APS	0.54	80.09	1892	3.29	7.2	300±10.4	2±0.30	64	0	0	E
	35	3987	APS	0.54	80.09	1892	2.44	7.2	309±6.2	1.7±0.17	47	0	0	E
	36	3987	APS	0.54	80.09	1892	2.44	7.2	321±7.6	1.7±0.20	69	0	0	E
13	37	4107	APM	−0.06	78.20	1423	2.44	7.06	295±11.3	2.7±0.23	55	0	0	E
	38	4111	APM	−0.08	78.14	1407	1.84	7.06	287±5.6	2.4±0.36	62	0	0	E
	39	4111	APM	−0.08	78.14	1407	1.82	7.06	309±7.2	2.4±0.26	63	0	0	E
14	40	4206	APM	−0.86	76.64	1035	1.82	7.18	271±7.6	1.9±0.17	64	0	0	E
	41	4221	APM	−0.53	76.40	1036	2.34	7.18	268±6.0	2.2±0.20	58	0	0	SE
	42	4221	APM	−0.53	76.40	1036	2.34	7.18	292±7.8	1.9±0.26	58	0	0	SE

Note: AL, Altitude; VT, Vegetation types; AT, Air temperature; RH, Relative humidity; PR, Precipitation; ST, Soil temperature; Eh, Redox potential; LI, Depth of litter; SL, Slope; CC, Canopy closure; CH, Canopy height. AS, Aspect; EBLF, evergreen broad-leaved forest; EDBF, mixed evergreen and deciduous broad-leaved forest; BLCF, mixed broadleaf-conifer forest; DCF, dark coniferous forest; APS, alpine shrubland; APM, alpine meadow; E, East; S, South; W, West, SE, Southeast; NW, Northwest.

### Statistical Analysis

For analyses of species richness we used the total species number per transect (n = 42). Coverage and biomass values were averaged in each transect and elevation.

All environmental variables measured on the quadrat scale were averaged in each transect. The generalized additive models (GAMs), a widely used biological nonparametric generalization of multiple linear regression, were used to describe responses of species richness, coverage, biomass and thickness of bryophyte to elevation change. In the GAMs, a link function is related to predictor variables by scatterplot smoothers instead of least-squares fits, and is subject to less restrictive distributional assumptions than multiple linear regression [46]. The GAMs analysis was implemented by *S*-plus 8.0 statistical software (Insightful Corporation, Seattle) [47]. Relationships between bryophyte biomass and number of species, cover, and thickness of bryophyte layer were established using simple regression analysis. The relationship between species composition and environmental variables (including altitude, air temperature, relative humidity (RH), precipitation, soil temperature, soil moisture, depth of litter, vegetation type, aspect, slope, canopy height and closure, soil pH and Eh) was evaluated using canonical correspondence analysis (CCA), with detrended correspondence analysis (DCA) used to obtain estimates of gradient lengths (in standard deviation (S.D.) units of species turnover) [48]. CCA is a good ordination method which can reflect the variation of biotic communities with environmental conditions or the response of biotic communities to environmental parameters [49]. DCA in our study revealed lengths of the gradient was longer than 4.0, therefore the unimodel should be selected against the liner modal method [49]. In the CCA analysis, the percentage cover of the dominant genus was used as the species input. The forward selection modus of a CCA [49] was implemented to rank the importance of each environmental variable, and to remove any environmental variables insignificantly contributing to the observed variation. Monte-Carlo tests with 1000 unrestricted permutations were performed to test the statistical significance of each environmental variable (at α = 0.05 to enter or stay in model) for the variance of bryophyte distribution. The CCA analyses were performed with CANOCO 4.5 [50].

## Results

### Species Composition

695 specimens were collected across all transects and identified to 165 species level, including 42 liverworts and 123 mosses, representing 64 genera within 30 bryophyte families (Table S1). The ground-layer bryophyte richness in different transects ranges from 7 to 26 species (Table 2). Generally, the number of mosses is higher than that of liverworts at each altitude (Table 2). According to percentage cover measurements, the most popular families are Amblystegiaceae (with 2 genus and 8 species), Brachytheciaceae (with 5 genera and 23 species), Grimmiaceae (with 3 genera and 9 species), Hylocomiaceae (with 4 genera and 5 species), Mniaceae (with 4 genera and 11 species) and Thuidiaceae (with 5 genera and 8 species).

**Table 2:** Number of bryophytes along the altitudinal gradient.

Site No.	Transect No.	Altitude (m a.s.l.)	Number of total Bryophytes	Number of Mosses	Number of Liverworts
1	1	2001	7	7	0
	2	2001	12	12	0
	3	2023	7	7	0
2	4	2301	21	19	2
	5	2301	19	18	1
	6	2359	25	24	1
3	7	2760	19	14	5
	8	2760	20	17	3
	9	2784	23	16	7
4	10	2964	26	19	7
	11	2964	27	16	11
	12	2964	19	14	5
5	13	3000	20	13	7
	14	3044	21	13	8
	15	3060	22	13	9
6	16	3060	14	13	1
	17	3103	11	10	1
	18	3106	7	7	0
7	19	3106	11	9	2
	20	3174	7	7	0
	21	3174	11	11	0
8	22	3174	19	14	5
	23	3247	18	13	5
	24	3247	18	15	3
9	25	3247	11	11	0
	26	3650	14	14	0
	27	3650	11	10	1
10	28	3650	7	7	0
	29	3725	14	13	1
	30	3758	24	17	7
11	31	3758	13	12	1
	32	3817	8	7	1
	33	3817	11	8	3
12	34	3817	9	9	0
	35	3987	8	7	1
	36	3987	11	9	2
13	37	3987	13	11	2
	38	4107	15	12	3
	39	4111	18	13	5
14	40	4111	21	19	2
	41	4206	15	14	1
	42	4221	20	19	2

### Distribution of Bryophytes along Altitudinal Gradient

**Figure 2:**
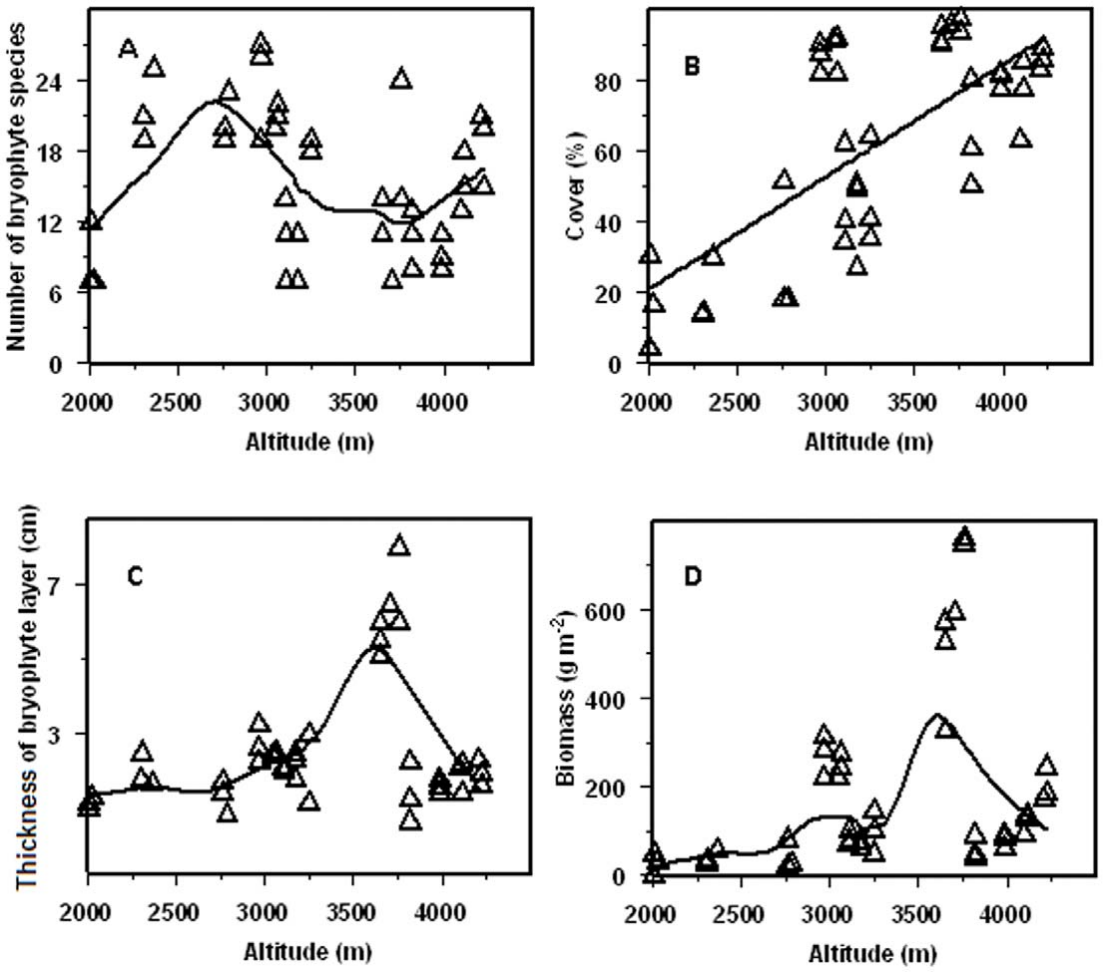
Distribution characters of terrestrial bryophyte along the altitudinal gradient. A, Number of bryophyte species; B, Cover of terrestrial bryophyte; C, Thickness of bryophyte layer; D, Biomass of terrestrial bryophyte.

A humped relationship between bryophyte species number and the elevation is clear below 3,650 m a.s.l., while an increasing trend of species number above 3,650 m a.s.l. were observed (*R  = 0.605, P  = 0.01*). Elevation trend of species richness is highly curvature as the elevation varies from 2,001 m to 4,221 m a.s.l. (Figure 2A). Along the altitude, bryophytes are respectively dominated in cover by (Table 3): Thuidium, Brachythecium, and Eurhynchium (2,001–2,359 m a.s.l.); Thuidium and Brachythecium, (2,760–2,784 m a.s.l.); Actinothuidium, Hylocomium, Pleurozium, and Rhizomnium (2,964–3060 m a.s.l.); Brachythecium and Eurhynchium (3,103–3,247 m a.s.l.); Drepanocladus, Racomitrium and Sanionia (3,650–3,758 m a.s.l.); Brachythecium (3,817 m a.s.l.); and Drepanocladus, Brachythecium and Sanionia (3,987–4,221 m a.s.l.).

**Table 3:** Forest types and the most-prevalent bryophyte genus along the altitudinal gradient.

Site No.	Altitude (m a.s.l.)	Dominant bryophyte genus	Forest types
1	2001–2023	Thuidium, Brachythecium, and Eurhynchium	Evergreen broad-leaved forest
2	2301–2359	Thuidium, Brachythecium, and Eurhynchium	Mixed evergreen and deciduous broad-leaved forest
3	2760–2784	Thuidium and Brachythecium	Mixed broadleaf-conifer forest
4	2964	Actinothuidium, Hylocomium, Pleurozium, and Rhizomnium	Dark coniferous forest
5	3044–3060	Actinothuidium, Hylocomium, Pleurozium, and Rhizomnium	Dark coniferous forest
6	3103–3106	Brachythecium and Eurhynchium	Dark coniferous forest
7	3174	Brachythecium and Eurhynchium	Dark coniferous forest
8	3247	Brachythecium and Eurhynchium	Dark coniferous forest
9	3650	Drepanocladus, Sanionia, and Racomitrium	Alpine shrubland
10	3725–3758	Drepanocladus, Sanionia, and Racomitrium	Alpine shrubland
11	3817	Brachythecium	Alpine shrubland
12	3987	Brachythecium Drepanocladus, and Sanionia	Alpine shrubland
13	4107–4111	Brachythecium Drepanocladus, and Sanionia	Alpine meadow
14	4206–4221	Brachythecium Drepanocladus, and Sanionia	Alpine meadow

Terrestrial bryophyte cover increases linearly from 17.4% to 95.6% with increasing elevation (*R  = 0.711, P<0.001*) (Figure 2B). The bryophyte layer is well-developed in the upper montane forests above 2,784 m a.s.l., with the highest mean coverage of 95.64% at 3,758 m a.s.l. On the contrary, bryophyte cover is low below 2784 m a.s.l., and is often inconspicuous at 2,001 m a.s.l., even all trunks and branches are covered with dense bryophyte cushions.

**Figure 3:**
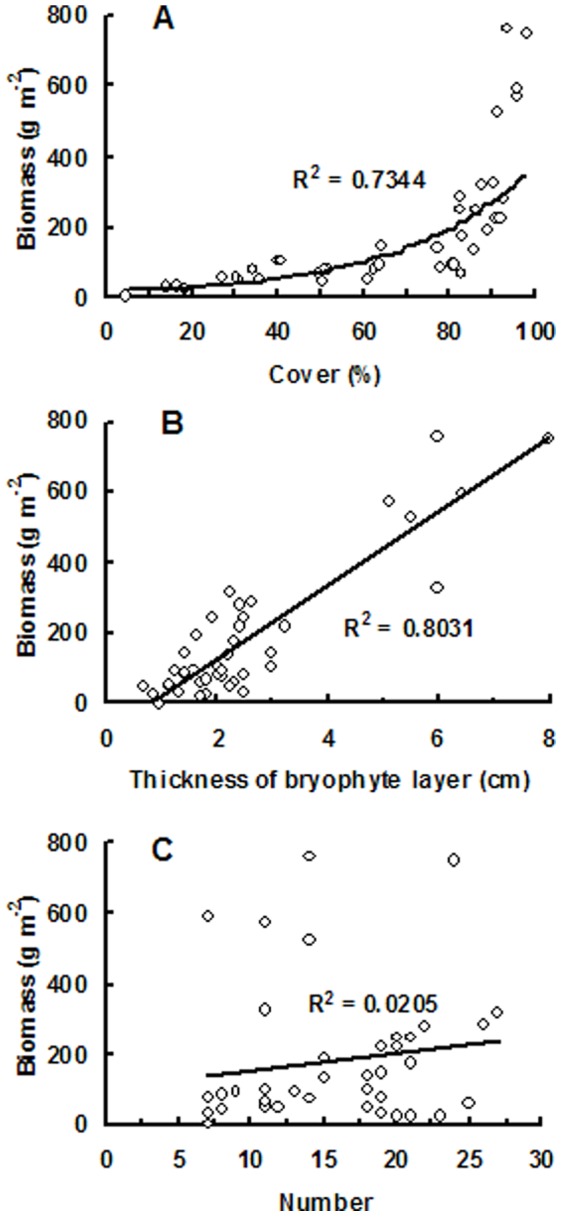
Correlation analysis between bryophyte biomass and A) cover, B) thickness of bryophyte layer, and C) species number.

A clear humped relationship is observed between the bryophyte thickness and biomass and the elevation, with a maximum thickness of 8 cm and an averaged biomass of 700.3 g m^−2^ around 3,758 m a.s.l. (Figure 2C, 2D). The biomass is very low (less than 50 g m^−2^) between 2,001 and 2,784 m a.s.l., where the ecotones are evergreen broad-leaved forest, mixed evergreen and deciduous broad-leaved forest, and mixed broadleaf-conifer forest (Table 3). Higher biomass always coincided with both higher cover and higher thickness of bryophyte layer (Figure 2). Regression analysis also indicates that a significant exponential-relationship and a linear correlation are separately existed between bryophyte biomass and cover (p<0.05, Figure 3A), and between bryophyte biomass and bryophyte layer thickness (p<0.05, Figure 3B). Bryophyte biomass is unrelated to species number (Figure 3C).

### Factors Influencing Bryophyte Species Composition and Distribution

**Figure 4:**
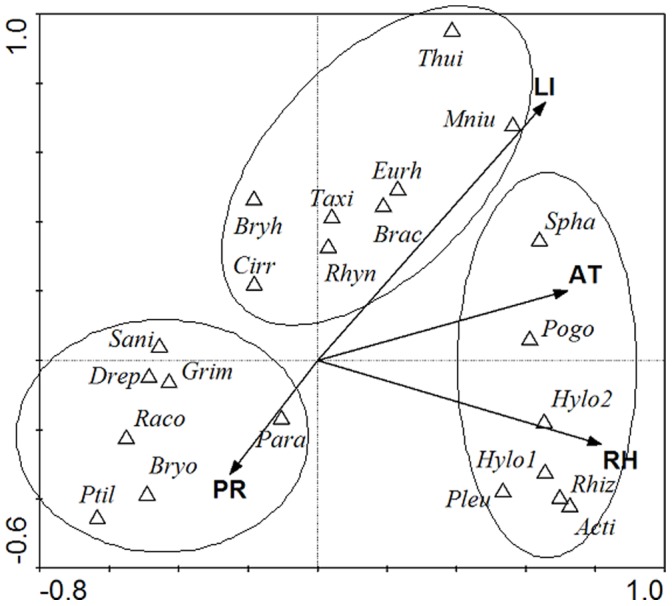
CCA ordination Bio-plot for the most abundance bryophyte species. Genus abbreviation: Acti = Actinothuidium, Brac = Brachythecium, Bryh = Bryhnia, Bryo = Bryonoguchia, Cirr = Cirriphyllum, Drep = Drepanocladus, Eurh = Eurhynchium, Grim = Grimmia, Hylo1 =  Hylocomiastrum, Hylo2 =  Hylocomium, Mniu = Mnium, Para = Paraleucobryum, Pleu = Pleurozium, Pogo = Pogonatum, Ptil = Ptilium, Raco = Racomitrium, Rhiz = Rhizomnium, Rhyn = Rhynchostegium, Sani = Sanionia, Spha = Sphagnum, Taxi = Taxiphyllum, Thui = Thuidium*;* Environment abbreviation: AT = Air temperature, LI = Depth of litter, PR = Precipitation, RH = Relative humidity.

According to CCA, depth of litter, air temperature, relative humidity and precipitation are the main factors correlated with bryophyte composition (Figure 4). The eigenvalues for the two first axes of the partial CCA are 0.720 and 0.395, respectively (Table 4). The first canonical axis explained 38.9%, the second 60.2%, and the first four axes 72.7% of the variation in species composition explained by the recorded explanatory variables. As shown in Figure 4, air temperature, and RH correlated to axis I, depth of litter and precipitation correlated with axis II. According to CCA, bryophytes can be categorized to 3 groups. For example, Actinothuidium, Hylocomiastrum, Hylocomium, Pleurozium, Pogonatum, Rhizomnium and Sphagnum are genus located in sites with higher RH and higher air temperature. Brachythecium, Bryhnia, Cirriphyllum, Eurhynchium, Mnium, Rhynchostegium, Taxiphyllum and Thuidium are genus growing in sites with higher litter depth. Bryonoguchia, Drepanocladus, Grimmia, Paraleucobryum, Ptilium, Racomitrium and Sanionia are genus growing in sites with higher precipitation.

**Table 4:** Results of the CCA, showing eigenvalues, cumulative explained variance of species data, species–environment correlation coefficients, and correlation coefficients of the environmental variables for the 4 axes established.

	Axes				F	P
	1	2	3	4		
Eigenvalues	0.720	0.395	0.143	0.089		
Species-environment correlations	0.969	0.875	0.864	0.737		
Cumulative percentage variance						
of species data	38.9	60.2	67.9	72.7		
of species-environment relation	53.4	82.7	93.4	100.0		
Environmental variables correlation coefficients						
AT	0.6982	0.176	0.5713	−0.0441	9.120	0.0010
RH	0.7924	−0.2129	0.2557	−0.3168	16.19	0.0010
PR	−0.2446	−0.2874	0.4881	−0.5261	15.54	0.0010
LI	0.6359	0.6515	−0.0199	0.0893	18.99	0.0010

The *F*-value and the significance of each independent environmental variable are also indicated.

## Discussion

There is rich ground bryophyte diversity in Gongga Mountain, where 165 bryophyte species, including 42 liverworts and 123 mosses are found. Regions in the southwest of China are always rich in bryophytes, for example 153 bryophyte species including 118 mosses and 35 liverworts were reported in Xiaozhaizi Nature Reserve [51], and 134 mosses were reported in Wanglang Nature Reserve [52]. Rather, bryophytes in Gongga Mountain contribute greatly to the overall plant biodiversity in this region, and, even in Southwest China. However, we just investigated ground bryophytes in this study, and broader investigations including other epiphytes types should be conducted later.

Although a clear humped relationship between the number of species and altitude below 3,650 m a.s.l., and an increasing trend above 3,650 m a.s.l. were observed, the elevation trend is highly curved from 2,001 m to 4,221 m a.s.l. This result differs significantly from those previous report in other places, where a decreasing trend [16], an increasing trend [12], [17], [18], or a hump-shaped distribution of species richness [15] were founded. In our study, the curved altitudinal trend of bryophyte species richness, especially the second increase trend following the first hump, is a new finding. In the present study, the humped relationship between species number and altitude stops at about 3,650 m a.s.l., the treeline of Gongga Mountain Forest. At this location, bryophyte community changes to genus dominated by Drepanocladus, Sanionia, and Racomitrium, accompanying with the abrupt change of vegetation community from forest to open alpine shrubland. For the second increase of bryophyte diversity above 3,650 m a.s.l., one possible interpretation is the greater capacity of bryophytes to tolerate extreme conditions and the pioneer strategy of many bryophytes [18]. It could also be a part of a second hump which might appear if extended to higher altitudes. However, no investigation was conducted above 4,221 m a.s.l. in this study due to difficulty in accessing such sites.

The bryophyte distribution seems to be influenced by the elevation, and the bryophyte cover increases linearly with the increasing elevation. Vegetation types alternate along the altitudinal gradient (Table 3). From 2,001 to 2,358 m a.s.l., the vegetation are evergreen broad-leaved forest, and mixed evergreen and deciduous broad-leaved forests, where the ground bryophyte cover is low. From 2,964 to 3,987 m a.s.l., where the averaged bryophyte cover is higher than 85% with dark coniferous forest and alpine shrubland, and becomes an important vegetation layer. The terrestrial bryophyte cover linearly relates to elevation increase in Gongga Mountain, which is in accordance with the findings in tropical rain forests [53] and the Southern Appalachian Mountains [19].

The bryophyte biomass is linearly correlated with the thickness of bryophyte layer, exponentially correlated with the cover, but not correlated with the species number. In spite of this, higher biomass just appears at sites with both higher cover and higher thickness of bryophyte layer in this study (Figure 2). This kind of relationship likely results from the difference of species composition at different altitudes. For instance, pleurocarpous or creeping bryophytes generally have lower volume than the same cover of their acrocarpous or erect opponents. Therefore, large cover does not always mean a large biomass. However, a deep bryophyte layer thickness is commonly associated with particular species groups that often have large cover, which therefore produce a high biomass. Our result is in accordance with that provided by Kuusipalo [54], who developed bryophyte biomass models with two factors of percentage cover and height.

Results of CCA analysis suggests that the depth of litter, the air temperature, the precipitation and the relative humidity are the main factors influencing bryophyte species composition. One reason for the important role of air temperature, precipitation and the relative humidity in influencing bryophyte distribution might be the poikilohydric properties of bryophytes [10]. This result is in accordance with findings of Porley and Hodgetts [27], who explained that bryophyte distribution is influenced primarily by macroclimatic factors, including rainfall and temperature. Some other studies, suggesting that moisture is an important growth determinant more limiting than nutrients to bryophyte productivity [55], and that air temperature causes moss cover deterioration [56], [57], also support our results.

The depth of litter is another major factor influencing terrestrial bryophyte distribution according to CCA results. Forests from 2,001 to 3,247 m a.s.l. are dominated by tall trees, where many rocks, fallen logs, branches, and twigs on the ground can grow the bryophytes [15]. Forests within the ranges of 2,001–2,358 m a.s.l. and 3,103–3,247 m a.s.l. are respectively dominated by tall broadleaves and conifers with dense undergrowth vegetation, where a thick layer of litter is formed. Bryophyte growth might be inhibited by shading and litter leachates [58]–[61]. Forests within the altitude range of 2,760–3,060 m a.s.l. are dominated by mixed broadleaved-conifer or conifers with few shrubs only, where fallen branches and twigs provide a favorable substrate for the inhabitation of bryophytes but without the limitation of litter thickness. At altitudes higher than 3,650 m a.s.l., close to the timber line (approximately 3,600 m in Gongga Mountain), fallen branches and twigs are rare. With increasing altitude, the vegetation changes gradually into alpine meadow. The herbaceous vegetation and its litter have good moisture-holding capacity and nutrient availability at this region, and therefore benefit for bryophyte species [62].

### Conclusions

Our investigations show the rich terrestrial bryophyte diversity in Gongga Mountain. A clear humped relationship between the amount of species and the elevation below 3,650 m a.s.l., as well as an increasing trend above 3,650 m a.s.l. are found. From 2,001 to 4,221 m a.s.l, the species richness increases in a high curvature trends. These features significantly differ from those of previous investigations, especially the second increase trends following the first hump in our investigation.

The cover of terrestrial bryophyte increases with elevation, while the biomass and the thickness of bryophyte exhibit a clear humped relationship with the elevation. The elevation of 3,758 m a.s.l. is a key point for bryophytes, where the averaged biomass of 700.3 g m^−2^ and the maximum thickness of 8 cm are observed. These results are helpful for modeling ecosystem carbon and nutrient cycling, as well as promoting the understanding of bryophytes’ ecological role in the forest ecosystem.

The bryophyte distribution is primarily associated with the depth of litter, the air temperature and the precipitation. The relationship between bryophyte distribution and climatic proxies might be useful for modeling responses of bryophyte to climate changes. However, in this study, just ground bryophytes were investigated, and no sites above 4,221 m a.s.l. were examined.

## Supporting Information

Table S1 (DOC)
