# Supplementary material for: Bryophyte Species Richness and Composition along an Altitudinal Gradient in Gongga Mountain, China
Source: PLoS One. 2013 Mar 5;8(3):e58131. doi: 10.1371/journal.pone.0058131 (PMC3589371; doi:10.1371/journal.pone.0058131)
Supplement: Table S1 — (DOC) [file pone.0058131.s001.doc]

**Appendix I.** List of species encountered in the study

| **Taxa** | **Genus** | **Family** | **Altitudinal range** |
| --- | --- | --- | --- |
| *Abietinella abietina* (Hedw.) Fleisch. | Abietinella | Thuidiaceae | 3750, 2964, 3060, 3100, 3170, 3247 |
| *Actinothuidium hookeri* (Mitt.) Broth. | Actinothuidium | Thuidiaceae | 2964, 3060, 3100, 3170, 3247 |
| *Anastrophyllum joergensenii* Schiffn. | Anastrophyllum | Lophoziaceae | 4100, 4220 |
| *Anastrophyllum minutum* (Schreb.) R.M.Schist. | Anastrophyllum | Lophoziaceae | 3750, 3820, 3987, 4100, 4220 |
| *Anastrophyllum saxicolum* (schrad.) schust. | Anastrophyllum | Lophoziaceae | 3750, 4100, 4220 |
| *Aneura pinguis* (L.) Dum. | Aneura | Aneuraceae | 3060 |
| *Apometzgeria pubescens (Schrand.) Kuwah.* | *Apometzgeria* | *Metzgeriaceae* | *3820, 3987, 4100, 4220* |
| *Bazzania yoshinagana* (steph.) steph. | Bazzania | Lepidoziaceae | 2964, 3100, 3247 |
| *Brachythecium albicans* (Hedw.) B.S.G. | Brachythecium | Brachytheciaceae | 2300 |
| *Brachythecium plumosum* (Hedw.) B.S.G. | Brachythecium | Brachytheciaceae | 4100, 4220 |
| *Brachythecium buchananii* (Hook.) Jaeg. | Brachythecium | Brachytheciaceae | 3100, 3247, 3820, 3987, 4100, 4220 |
| *Brachythecium campylothallum* C. Muell. | Brachythecium | Brachytheciaceae | 2000, 2300 |
| *Brachythecium erythrorhizon* B.S.G. | Brachythecium | Brachytheciaceae | 2000, 2300 |
| *Brachythecium kuroishicum* Besch. | Brachythecium | Brachytheciaceae | 3100, 3170, 3247, 3820, 4220 |
| *Brachythecium moriense* Besch. | Brachythecium | Brachytheciaceae | 2300, 2750 |
| *Brachythecium piligerum* Card. | Brachythecium | Brachytheciaceae | 2300, 2750 |
| *Brachythecium populeum (Hedw.) B.S.G.* | Brachythecium | Brachytheciaceae | 2300 |
| *Brachythecium pulchellum* Broth. | Brachythecium | Brachytheciaceae | 3650 |
| *Brachythecium rotaeanum* De Not. | Brachythecium | Brachytheciaceae | 2000, 2300 |
| *Brachythecium rutabulum (Hedw.) B.S.G.* | Brachythecium | Brachytheciaceae | 2300, 2750, 3987 |
| *Brachythecium thraustum* C. Muell. | Brachythecium | Brachytheciaceae | 2300, 3820 |
| *Breidleria pratensis* (Koch ex Spruce) Loeske | Breidleria | Hypnaceae | 3750, 4220 |
| *Brotherella falcata* (Dozy et Molk.) Fleisch. | Brotherella | Sematophyllaceae | 2300 |
| *Brotherella henonii* (Duby) Fleisch. | Brotherella | Sematophyllaceae | 2300, 2750 |
| *Bryhnia novae-angliae* (Sull. et Lesq.) Grout | Bryhnia | Brachytheciaceae | 2000, 2300, 3170, 3820, 3987, 4220 |
| *Bryonoguchia molkenboeri* (Lac.) Iwats. et Inoue | Bryonoguchia | Thuidiaceae | 3247, 3750 |
| *Bryum argenteum* Hedw. | Bryum | Bryaceae | 3650 |
| *Bryum caespiticium* Hedw. | Bryum | Bryaceae | 2000, 2300, 2750, 2964 |
| *Calypogeia fissa* (L.) Raddi | Calypogia | Calypogeiaceae | 3060 |
| *Calypogeia muelleriana* (Schiffn.) K. Muell. | Calypogia | Calypogeiaceae | 2964 |
| *Campylopus alpigena* Broth. | Campylopus | Dicranaceae | 2960, 3060 |
| *Campylopus flexosus* (Hedw.) Brid. | Campylopus | Dicranaceae | 2964, 3060, 3100 |
| *Campylopus umbellatus* (Arnoth.) Par. | Campylopus | Dicranaceae | 2,964 |
| *Chiloscyphus coadunatus* (Sw.) RM Schust. & JJ Engel | Chiloscyphus | Geocalycaceae | 3247 |
| *Chiloscyphus latifolius* (Nees) JJ Engel & RM Schust. | Chiloscyphus | Geocalycaceae | 3060, 3820 |
| *Chiloscyphus magellanicus* Steph. | Chiloscyphus | Geocalycaceae | 3987 |
| *Chiloscyphus polyanthos* var. rivularis | Chiloscyphus | Geocalycaceae | 3247 |
| *Chiloscyphus profundus* (Nees) J.J. Engel et R.M. Schust. | Chiloscyphus | Geocalycaceae | 2964, 3820 |
| *Cirriphyllum piliferum* (Hedw.) Grout | Cirriphyllum | Brachytheciaceae | 2300, 2750, 3174, 3817, 3987, 4100, 4220 |
| *Climacium dendroides* Web. et Mohr | Climacium | Climaciaceae | 3650 |
| *Climacium japonicum* Lindb. | Climacium | Climaciaceae | 2750 |
| *Ctenidium lychnites* (Mitt.) Broth. | Ctenidium | Hypnaceae | 2300, 2964, 3100 |
| *Dicranodontium asperulum* (Mitt.) Broth. | Dicranodontium | Dicranaceae | 2360, 2964 |
| *Dicranodontium denudatum* (Brid.) Britt. | Dicranodontium | Dicranaceae | 2360, 3060 |
| *Dicranodontium uncinatum* (Harv.) Jaeg. | Dicranodontium | Dicranaceae | 2964, 3060, 3247 |
| *Dicranum bonjeanii* De Not. | Dicranum | Dicranaceae | 3987 |
| *Dicranum drummondii* C. Muell. | Dicranum | Dicranaceae | 2964, 3750 |
| *Dicranum elongatum var. var. sphagni T. Jens.* | Dicranum | Dicranaceae | 3750 |
| *Dicranum flagilifolium* Lindb. | Dicranum | Dicranaceae | 2750, 2964 |
| *Dicranum assamicum* Dix. | Dicranum | Dicranaceae | 2964 |
| *Dicranum japonicum* Mitt. | Dicranum | Dicranaceae | 2750, 3060 |
| *Dicranum majus* Turn. | Dicranum | Dicranaceae | 2964, 3060 |
| *Dicranum polysetum* Sw. | Dicranum | Dicranaceae | 3750 |
| *Dicranum scoparium* Hedw. | Dicranum | Dicranaceae | 2964, 3060, 3100, 3750, 4100, 4220 |
| *Didymodon cordatus* Jur. | Didymodon | Pottiaceae | 3987, 4100, 4220 |
| *Didymodon ferrugineus* (Schimp. ex Besch.) Hill | Didymodon | Pottiaceae | 3650, 3987, 4100, 4220 |
| *Didymodon rigidulus* Hedw. | Didymodon | Pottiaceae | 4100, 4220 |
| *Didymodon vinealis* (Brid.) R.H. Zander | Didymodon | Pottiaceae | 3650, 3750 |
| *Drepanocladus aduncus* (Hedw.) Warnst. | Drepanocladus | Amblystegiaceae | 3650, 3750, 3820, 3987, 4100, 4220 |
| *Drepanocladus trichophyllus* (Warnst.) Podp. | Drepanocladus | Amblystegiaceae | 3650, 3750, 4100, 4220 |
| *Drepanocladus intermedius* (Lindb.) Warnst. | Drepanocladus | Amblystegiaceae | 3650, 3750, 4220 |
| *Drepanocladus revolvens* (Sw.) Warnst. | Drepanocladus | Amblystegiaceae | 3750, 4100, 4220 |
| *Drepanocladus sendtneri* (Schimp.) Warnst. | Drepanocladus | Amblystegiaceae | 3650, 3750 |
| *Drepanocladus sordidus* (Müll. Hal.) Hedenas | Drepanocladus | Amblystegiaceae | 3750, 4220 |
| *Drepanocladus vernicosus* (Mitt.) Warnst. | Drepanocladus | Amblystegiaceae | 3750, 4100, 4220 |
| *Eurhynchium angustirete* (Broth.) T. Kop. | Eurhynchium | Brachytheciaceae | 3820, 3987, 4220 |
| *Eurhynchium arbuscula* Broth. | Eurhynchium | Brachytheciaceae | 2300, 2750 |
| *Eurhynchium kirishimense* Takaki | Eurhynchium | Brachytheciaceae | 2000, 3820, 4100, 4220 |
| *Eurhynchium praelongum* (Hedw.) Schimp. | Eurhynchium | Brachytheciaceae | 2000, 2300 |
| *Eurhynchium savatieri Schimp. ex Besch.* | Eurhynchium | Brachytheciaceae | 2000, 2300, 2750, 3100, 3170, 3247, 3820, 3987,4220 |
| *Fossombronia angulosa* (dicks.) raddi | Fossombronia | Fossombronia | 2964 |
| *Girgensohnia ruthenica* (Weinm.) Kindb. | Girgensohnia | Climaciaceae | 2964 |
| *Grimmia longirostris* Hook. (Grimmia affinis Hornsch.) | Grimmia | Grimmiaceae | 3650 |
| *Grimmia pilifera* P. Beauv. | Grimmia | Grimmiaceae | 3650, 3750, 4100, 4220 |
| *Helodium blandowii* (Web. & Mohr) Warnst. | Helodium | Thuidiaceae | 3750, 4100, 4220 |
| Hylocomiastrum himalayanum (Mitt.) Broth. | Hylocomiastrum | Hylocomiaceae | 2964, 3060, 3247 |
| *Hylocomiastrum pyrenaicum* (Spruce) Fleisch. ex Broth. | Hylocomiastrum | Hylocomiaceae | 3650 |
| *Hylocomium splendens* (Hedw.) B.S.G. | Hylocomium | Hylocomiaceae | 2964, 3060, 3100, 3247, 3650 |
| *Hynum oldhamii* (Mitt.) Jaeg. | Hypnum | Hypnaceae | 2964, 3100 |
| *Hypnum circinale* Hook. | Hypnum | Hypnaceae | 2750 |
| *Hypnum hamulosum* B.S.G. | Hypnum | Hypnaceae | 2964, 3060 |
| *Hypnum revovlutum* (Mitt.) Lindb. | Hypnum | Hypnaceae | 4220 |
| *Hypopterygium fauriei* Besch. | Hypopterygium | Hypopterygiaceae | 2300 |
| *Lepidozia reptans* (L.) Dum. | Lepidozia | Lepidoziaceae | 2750, 2964, 3060, 3170, 3247 |
| *Lophozia setosa* (Mitt.) Steph. | Lophozia | Lophoziaceae | 2750, 3060 |
| *Lophozia incisa* (Schrad.) Dum. | Lophozia | Lophoziaceae | 2964 |
| *Lophozia ascendens* (Warnst.) Shust. | Lophozia | Lophoziaceae | 3700, 3820 |
| *Metzgeria consanguinea* Schiffn. | Metzgeria | Metzgeriaceae | 2300 |
| *Mnium laevinerve* Card. | Mnium | Mniaceae | 2964, 3060, 3247 |
| *Mnium lycopodioides* Schwacgr. | Mnium | Mniaceae | 2300, 2750, 2964, 3060, 3247 |
| *Mnium undulatum* var. var. densirete Broth. | Mnium | Mniaceae | 2300 |
| *Oncophorus wahlenbergii* Brid. | Oncophorus | Dicranaceae | 2964, 3060 |
| *Pallavicinia subciliata* (Aust.) Steph. | Pallavicinia | Pallavicineaceae | 3060 |
| *Paraleucobryum enerve* (Thed.) Loesk. | Paraleucobryum | Dicranaceae | 3060, 3650, 3750, 4100, 4220 |
| *Paraleucobryum longifolium* (Hedw.) Loesk. | Paraleucobryum | Dicranaceae | 2750, 3100, 3247, 4100, 4220 |
| *Philonotis fontana* Brid. | Philonotis | Bartramiaceae | 3820, 3987, 4100, 4220 |
| *Plagiochila asplenioides* (L.) Dumotier. | Plagiochila | Plagiochilaceae | 2750, 2964 |
| *Plagiochila bifaria* (Sw.) Lindenb. | Plagiochila | Plagiochilaceae | 2750, 3750 |
| *Plagiochila Killarniensis* Pears. | Plagiochila | Plagiochilaceae | 3060 |
| *Plagiochila perserrata* Herz. | Plagiochila | Plagiochilaceae | 2964, 3170 |
| *Plagiochila porelloides* (Torrey ex Nees) Lindenb. | Plagiochila | Plagiochilaceae | 2750 |
| *Plagiochila pulcherrima* Horik. | Plagiochila | Plagiochilaceae | 2750 |
| *Plagiochila spinulosa* (Dicks.) Dumort. | Plagiochila | Plagiochilaceae | 2964, 3247 |
| *Plagiochila delavayi* Steph. | Plagiochila | Plagiochilaceae | 3060 |
| *Plagiochila deltoidea Lindenb.* | Plagiochila | Plagiochilaceae | 2964 |
| *Plagiomnium vesicatum* (Besch.) T. Kop. | Plagiomnium | Mniaceae | 2000, 2300, 2964, 3060, 3247, 3820, 3987, 4220 |
| *Plagiomnium maximoviczii* (Lindb.) T.Kop. | Plagiomnium | Mniaceae | 2750, 3060 |
| *Plagiomnium ellipticum* (Brid.) T. Kop. | Plagiomnium | Mniaceae | 2000, 2300, 2750, 2964, 3170, 3247 |
| *Plagiothecium cavifolium* (Brid.) Iwats. | Plagiothecium | Plagiotheciaceae | 2300, 2750, 3170 |
| *Plagiothecium curvifolium* Schlieph. ex Limpr. | Plagiothecium | Plagiotheciaceae | 2000, 2300 |
| *Plagiothecium denticulatum* (Hedw.) B.S.G. | Plagiothecium | Plagiotheciaceae | 2750, 3060 |
| *Plagiothecium neckeroideum* B.S.G. | Plagiothecium | Plagiotheciaceae | 2300, 2964, 3060, 3170, 3247 |
| *Plagiothecium nemorale* (Mitt.) Jaeg. | Plagiothecium | Plagiotheciaceae | 2000, 2300 |
| *Plagiothecium piliferum* (Hartm) B.S.G. | Plagiothecium | Plagiotheciaceae | 2300, 3820 |
| *Plagiothecium platyphyllum* Moenk. | Plagiothecium | Plagiotheciaceae | 3247 |
| *Plagiothecium succulentum* (Wils.) Lindb. | Plagiothecium | Plagiotheciaceae | 2000, 2300, 3100 |
| *Pleurozium schreberi* (Brid.) Mitt. | Pleurozium | Hylocomiaceae | 2750, 2964, 3060, 3247, 3650, 3820, 3987, 4100, 4220 |
| *Pogonatum cirratum* (Sw.) Brid. | Pogonatum | Polytrichaceae | 2964, 3247 |
| *Pogonatum contortum* (Brid.) Lesq. | Pogonatum | Polytrichaceae | 3170 |
| *Pogonatum fastigiatum* Mitt. | Pogonatum | Polytrichaceae | 2750, 2964 |
| *Pogonatum neesii* (C. Muell.) Dozy. | Pogonatum | Polytrichaceae | 2964, 3100, 3170 |
| *Pogonatum urnigerum* (Hedw.) P. Beauv. | Pogonatum | Polytrichaceae | 3650 |
| *Polytrichastrum alpinum* (Hedw.) G. Smith | Polytrichastrum | Polytrichaceae | 3100, 3170, 3247, 3750 |
| *Polytrichastrum formosum* var. var. densifolium (Hedw.) G. Sm. | Polytrichastrum | Polytrichaceae | 3100, 3170 |
| *Porella arboris-vitae* (With.) Grolle | Porella | Porellaceae | 2300 |
| *Porella canariensis* (F. Weber) Underw. | Porella | Porellaceae | 3750, 4100, 4220 |
| *Porella obtusata* var. macroloba (Steph.) Hatt. et Zhang | Porella | Porellaceae | 3650, 3750, 4100 |
| *Porella pinnata* L&amp. | Porella | Porellaceae | 3060 |
| *Ptilium crista-castrensis* (Hedw.) De Not. | Ptilium | Hypnaceae | 3650, 3750 |
| *Racomitrium barbuloides* Card. | Racomitrium | Grimmiaceae | 3750, 3987, 4100, 4220 |
| *Racomitrium angustifolium* Broth. | Racomitrium | Grimmiaceae | 3650, 3750, 4100, 4220 |
| *Racomitrium ericoides* (Hedw.) Brid. | Racomitrium | Grimmiaceae | 3650, 4220 |
| *Racomitrium heterostichum* (Hedw.) Brid. | Racomitrium | Grimmiaceae | 3650, 3750, 4220 |
| *Racomitrium japonicum* Dozy & Molk. | Racomitrium | Grimmiaceae | 3650, 3750, 3820, 3987, 4100, 4220 |
| *Racomitrium laetum* Besch. et Card. | Racomitrium | Grimmiaceae | 3750 |
| *Rhizomnium hattorii* T.Kop. | Rhizomnium | Mniaceae | 2300, 2750, 2964, 3060, 3170, 3240 |
| *Rhizomnium nudum* (Britt. et Willioms) T.Kop. | Rhizomnium | Mniaceae | 2964, 3060 |
| *Rhizomnium punctatum* (Hedw.) T.Kop. | Rhizomnium | Mniaceae | 2964, 3060, 3100 |
| *Rhizomnium tuomikoskii* T. Kop. | Rhizomnium | Mniaceae | 2300, 2750 |
| *Rhodobryum roseum* (Hedw) Limpr. | Rhodobryum | Bryaceae | 2300, 2750 |
| *Rhynchostegium confertum* (Dicks.) Schimp. | Rhynchostegium | Brachytheciaceae | 2000, 3100, 3170, 3247, 3820, 3987, 4100, 4220 |
| *Rhynchostegium inclinatum* (Mitt.) Jaeger. | Rhynchostegium | Brachytheciaceae | 2000, 2300 |
| *Rhynchostegium tenuifolium* Reichardt | Rhynchostegium | Brachytheciaceae | 2000, 2300, 2750, 3247 |
| *Rhytidiadelphus squarrosus* (Hedw.) Warnst. | Rhytidiadelphus | Hylocomiaceae | 3247, 3820, 3987, 4100, 4220 |
| *Riccardia flagelifrons* Gao | Riccardia | Aneuraceae | 3247 |
| *Sanionia uncinata* (Hedw.) Loeske. | Sanionia | Amblystegiaceae | 3650, 3750, 3820, 3987, 4100, 4220 |
| *Scapania aspera* Bernet et M. Bernet | Scapania | Scapaniaceae | 2964, 3060 |
| *Scapania ciliata* S. Lac. | Scapania | Scapaniaceae | 2750 |
| *Scapania gracilis* (Lindb.) Kaal. | Scapania | Scapaniaceae | 4100 |
| *Scapania irrigua* (Nees) Dumort. | Scapania | Scapaniaceae | 2964 |
| *Scapania nemorea* (L.) Grolle | Scapania | Scapaniaceae | 2964 |
| *Scapania stephanii* K. Müll. | Scapania | Scapaniaceae | 4100 |
| *Scapania subnimbosa* Steph. | Scapania | Scapaniaceae | 2750, 3060, 3247, 3750 |
| *Scapania undulata* var. paludosa | Scapania | Scapaniaceae | 2750 |
| *Schistidium trichodon* (Brid.) Poelt. | Schistidium | Grimmiaceae | 3750, 4100, 4220 |
| *Sphagnum squarrosum* Crome | Sphagnum | Sphagnaceae | 2750, 3100, 3247 |
| *Sphagnum tenellum* Ehrh. Ex Hoffm | Sphagnum | Sphagnaceae | 2750 |
| *Taxiphyllum taxirameum* (Mitt.) Fleisch. | Taxiphyllum | Hypnaceae | 3170, 3820, 3987 |
| *Thamnobryum alopecurus* B.S.G. | Thamnobryum | Thamnobryaceae | 2750 |
| *Thuidium cymbifolium* (Dozy et Molk.) Dozy et Molk. | Thuidium | Thuidiaceae | 2000, 2300, 2750, 2964, 3247 |
| *Thuidium submicropteris* Card. | Thuidium | Thuidiaceae | 2960, 3060 |
| *Thuidium kanedae* Sak. | Thuidium | Thuidiaceae | 2300, 2750, 3100, 3170, 3750, 3987, 4220 |
| *Thuidium tamariscinum* (Hedw.) Schimp. | Thuidium | Thuidiaceae | 2000, 2300 |
| *Trachycystis flagellaris* (Sull. et Lesq.) Lindb. | Trachycystis | Mniaceae | 2964, 3060 |
| *Trichocolea tomentella* (Ehrb.) Dum. | Trichocolea | Trichocoleaceae | 2750, 2964, 3060, 3247 |
